# Absence of Documented Standard Modifiable Cardiovascular Risk Factors Among Catheterized Admissions in Bangladesh: Frequency and Coronary Angiographic Phenotype in a 20‐Year Registry Study

**DOI:** 10.1002/hsr2.73466

**Published:** 2026-11-04

**Authors:** Md Mahbub Hasan, M. Maksumul Haq, CM Shaheen Kabir, Mashhud Zia Chowdhury, Saidur Rahman Khan, Md. Hasanur Rahman, Sahela Nasrin, Isha Abdullah Ali, Khondoker Md Ferdoush Siraj, Arifur Rahman, Sayem Bin Latif

**Affiliations:** ^1^ Department of Cardiology Ibrahim Cardiac Hospital & Research Institute Dhaka Bangladesh

**Keywords:** Bangladesh, coronary angiography, coronary artery disease, SMuRF‐less, standard modifiable cardiovascular risk factors, ST‐elevation myocardial infarction

## Abstract

**Background and Aims:**

Coronary artery disease may occur without standard modifiable cardiovascular risk factors (SMuRFs), but the frequency and angiographic phenotype of such presentations in South Asian catheterization cohorts remain uncertain. We estimated registry‐defined SMuRF‐less frequency, tested alternative dyslipidaemia definitions, and compared angiographic phenotypes by SMuRF status.

**Methods:**

We conducted a retrospective, single‐centre, registry‐based cross‐sectional analysis of 51,864 admissions with matched catheterization records at a Bangladeshi tertiary cardiac centre from 2005 to 2024. Admissions were classified as SMuRF‐less when no qualifying hypertension, diabetes, smoking, or dyslipidaemia was documented or identified; dyslipidaemia reflected a documented diagnosis or qualifying lipid measurement. Documented‐only and statin‐inclusive definitions were sensitivity analyses. We calculated unadjusted prevalence ratios (PRs) and 95% confidence intervals (CIs).

**Results:**

Under the primary definition, 3172 admissions (6.1%) were SMuRF‐less, compared with 6.5% under the documented‐only definition and 1.6% under the statin‐inclusive definition. Among admissions with a classifiable angiographic phenotype, SMuRF‐less admissions more frequently had normal coronary arteries (24.9% vs. 10.7%; PR, 2.34; 95% CI, 2.18–2.50) and less frequently had obstructive coronary artery disease (56.6% vs. 71.2%; PR, 0.79; 95% CI, 0.77–0.82). Among admissions with obstructive disease, multivessel disease was less frequent (49.6% vs. 57.8%; PR, 0.86; 95% CI, 0.82–0.90), whereas recorded intracoronary thrombus was more frequent (5.3% vs. 3.2%; PR, 1.65; 95% CI, 1.33–2.04). Of 6635 ST‐elevation myocardial infarction admissions, 355 (5.4%) were SMuRF‐less; 318 (89.6%) had obstructive disease.

**Conclusion:**

Registry‐defined SMuRF‐less admissions, reflecting absence of documented qualifying factors rather than prospectively verified biological absence, were uncommon, and their estimated frequency depended on risk‐factor ascertainment. Although their recorded angiographic burden was generally lower, obstructive disease remained common, particularly in ST‐elevation myocardial infarction. The absence of documented SMuRFs should not provide clinical reassurance. Prospective patient‐level studies with standardized risk‐factor assessment and longitudinal follow‐up are needed.

## 1 Introduction

Ischaemic heart disease remains the leading cause of death worldwide. Its prevention and clinical risk assessment are centred largely on identifying and treating hypertension, diabetes mellitus, dyslipidaemia and smoking; the four standard modifiable cardiovascular risk factors (SMuRFs) [1, 2]. Although these factors are powerful and actionable determinants of cardiovascular risk, they do not encompass every pathway leading to coronary atherosclerosis or acute myocardial infarction (MI). Consequently, the absence of these factors should not be equated with the absence of either cardiovascular risk or underlying disease [3].

A growing literature has examined individuals who present with coronary artery disease (CAD) or acute coronary syndrome (ACS) without any identified SMuRF [4, 5]. A meta‐analysis of 15 studies involving 1,285,722 patients with ACS estimated that 11.6% were SMuRF‐less and reported higher risks of in‐hospital mortality and cardiogenic shock than among patients with at least one SMuRF [6]. Excess early mortality was also observed among SMuRF‐less patients with first‐presentation ST‐elevation myocardial infarction (STEMI) in the SWEDEHEART registry, giving rise to the proposed “SMuRF‐less paradox” [7]. The association is not uniform across clinical settings, however. In the CLARIFY registry, SMuRF‐less patients with established stable CAD experienced more favourable 5‐year outcomes than their SMuRF‐positive counterparts [8]. These contrasting findings indicate that SMuRF‐less status encompasses a heterogeneous population and that its implications depend on clinical presentation, risk‐factor ascertainment, treatment and duration of follow‐up.

These uncertainties are particularly relevant to South Asia. Participants from South Asian countries in the INTERHEART study experienced a first acute MI at a younger age than participants from other regions, while a multiethnic Asian registry demonstrated heterogeneity in the prognostic implications of SMuRF‐less status across ethnic groups [9, 10]. Nevertheless, most published studies have focused on selected ACS or first‐presentation MI populations and their clinical outcomes. The frequency and coronary angiographic phenotype of admissions classified as SMuRF‐less across a broader catheterization population remain less clearly described, particularly in Bangladesh.

An additional challenge is that SMuRF‐less status in routinely collected data usually denotes the absence of a documented factor rather than a prospectively verified biological absence. Incomplete recording of smoking or lipid measurements may therefore inflate the apparent SMuRF‐less proportion, whereas treating secondary‐prevention statin use as evidence of pre‐existing dyslipidaemia may substantially reduce it. Meaningful interpretation consequently requires both an explicit risk‐factor definition and transparent assessment of how alternative definitions affect the estimated frequency.

We therefore conducted a retrospective, registry‐based cross‐sectional analysis of catheterized admissions at a tertiary cardiac centre in Bangladesh between 2005 and 2024. The primary objectives were to estimate the proportion of admissions classified as SMuRF‐less under a primary registry‐based definition and to compare coronary angiographic phenotypes between SMuRF‐less and SMuRF‐positive admissions. Secondary objectives were to assess how alternative definitions of dyslipidaemia influenced the estimated SMuRF‐less frequency and to examine STEMI‐specific findings and calendar‐year distributions as exploratory analyses. Because longitudinal outcome data were unavailable, the study was designed to characterize frequency and coronary anatomy rather than to test the prognostic SMuRF‐less paradox.

## 2 Methods

### 2.1 Study Design, Setting and Reporting

We conducted a retrospective, single‐centre, registry‐based cross‐sectional analysis at a high‐volume tertiary cardiac care centre in Bangladesh. The study covered hospital admissions occurring between January 2005 and December 2024. Reporting was guided by the Strengthening the Reporting of Observational Studies in Epidemiology (STROBE) statement and the REporting of studies Conducted using Observational Routinely‐collected health Data (RECORD) extension [11, 12].

### 2.2 Data Sources and Cohort Assembly

The institutional electronic discharge database contained 108,520 admissions during the study period. Admission records were linked at the admission level to corresponding records in the institution's cardiac catheterization registry. Clinical diagnoses and recorded cardiovascular risk factors were obtained from the discharge database, while angiographic and procedural variables were obtained from the catheterization registry when a matching procedure record was available.

Eligible records were hospital admissions occurring between January 2005 and December 2024 with a corresponding matched record in the institutional cardiac catheterization registry. All 51,864 eligible matched admissions were included in the analytic cohort; admissions without a matched cardiac catheterization record were excluded. No additional exclusion criterion was applied within the matched cohort. Because all eligible matched records were included, no formal sample‐size calculation or sampling procedure was undertaken.

The registry spanned two data‐management systems: a legacy system used until the transition in 2014 and a successor system used from that transition through 2024. Documentation completeness differed between the systems; therefore, calendar‐year findings were treated as descriptive and were not interpreted as evidence of an epidemiological time trend.

The available analysis was structured at the admission level and did not establish that repeat admissions belonging to the same individual had been removed. Accordingly, the admission, rather than a confirmed unique patient, was the unit of analysis, and inferential tests treated admissions as independent observations.

### 2.3 Definition of SMuRF Status

The four standard modifiable cardiovascular risk factors (SMuRFs) were hypertension, diabetes mellitus, dyslipidaemia and smoking, consistent with the conventional SMuRF construct used in previous studies [3, 4, 5]. Family history of premature coronary artery disease was not included in the SMuRF definition. The SMuRF construct was limited to these four conventional factors and was not intended to encompass all potentially modifiable cardiovascular exposures. Body mass index was analysed separately as a non‐defining characteristic; obesity, physical inactivity, dietary factors and tobacco exposures not represented by the recorded smoking field were not incorporated into SMuRF classification. Hypertension and diabetes were identified from the corresponding pre‐existing registry diagnosis fields, and smoking from the registry smoking field.

A factor not recorded as present was treated as absent for the purpose of registry‐based classification. Consequently, “SMuRF‐less” indicates the absence of a documented qualifying risk factor and should not be interpreted as prospectively verified biological absence.

Dyslipidaemia was determined using three definitions. The prespecified primary definition classified dyslipidaemia as either a documented dyslipidaemia diagnosis or at least one available lipid measurement recorded for the admission that met any of the following thresholds: total cholesterol > 200 mg/dL, low‐density lipoprotein cholesterol > 130 mg/dL, triglycerides > 150 mg/dL, or high‐density lipoprotein cholesterol < 40 mg/dL. Missing lipid measurements were not imputed.

A documented‐only definition, based exclusively on the recorded dyslipidaemia flag, was examined to show the effect of incorporating measured lipid values. A second sensitivity definition additionally treated a recorded discharge prescription for a statin or other lipid‐lowering therapy as evidence of dyslipidaemia. Because lipid‐lowering treatment may represent secondary prevention rather than pretreatment dyslipidaemia, the statin‐inclusive definition was regarded as a deliberately conservative lower bound for SMuRF‐less prevalence and was not used for the principal comparisons.

An admission was classified as SMuRF‐less when none of the four SMuRFs was identified under the relevant definition and as SMuRF‐positive when at least one was identified. The primary lipid‐augmented definition was used for all phenotype comparisons.

### 2.4 Clinical and Angiographic Variables

Recorded non‐defining clinical characteristics included age, sex, body mass index, and chronic kidney disease. ST‐elevation myocardial infarction (STEMI) was identified from the discharge diagnosis. Left ventricular ejection fraction (LVEF) was obtained from the recorded echocardiographic assessment, and reduced left ventricular systolic function was defined as an LVEF of 40% or lower.

Coronary anatomy was classified using the angiographic grades recorded in the catheterization registry. Severe left‐main disease was defined as stenosis of at least 50%, while severe disease in the left anterior descending, left circumflex, or right coronary artery was defined as stenosis of at least 70%.

The overall coronary phenotype was classified as normal coronary arteries, non‐obstructive disease, or obstructive coronary artery disease. Non‐obstructive disease comprised recorded mild or moderate disease below the obstructive thresholds. Obstructive disease comprised severe involvement of at least one major epicardial coronary artery or the left main coronary artery. Myocardial bridging, congenital coronary artery anomalies, and spontaneous coronary artery dissection were separately identifiable in the catheterization registry and were not used to define the normal, non‐obstructive, or obstructive atherosclerotic coronary phenotypes. Admissions with these abnormalities were classified according to the recorded atherosclerotic stenosis burden in the major epicardial coronary arteries.

Among admissions with obstructive disease, its extent was reported using the registry categories of single‐, double‐, or triple‐vessel disease. Multivessel disease was defined as the combined presence of double‐ or triple‐vessel disease. Admissions with obstructive disease that were not assigned to one of these vessel‐count categories were retained in the obstructive‐disease denominator and reported separately as unassigned.

Additional registry‐recorded features included left‐main involvement, severe disease in each major epicardial coronary artery, chronic total occlusion, intracoronary thrombus, and coronary calcification. These features were analysed as recorded; angiographic images were not independently re‐adjudicated for the present analysis.

### 2.5 Study Measures and Analysis Populations

The principal study measures were the proportion of admissions classified as SMuRF‐less under the primary definition and the distribution of coronary angiographic phenotypes according to SMuRF status. The influence of the two alternative dyslipidaemia definitions on estimated SMuRF‐less prevalence was assessed as a sensitivity analysis.

Prevalence was examined overall and across five reported procedural categories: coronary angiography alone, coronary angiography and percutaneous coronary intervention during the same admission, staged angiography and intervention, percutaneous coronary intervention alone, and primary percutaneous coronary intervention. Admissions that could not be assigned to one of these categories were retained as “procedural category not otherwise classified.” The diagnosis‐based STEMI subgroup overlapped these procedural categories and was analysed separately.

Overall angiographic‐phenotype comparisons were restricted to admissions with a classifiable phenotype. Analyses of vessel count, individual coronary involvement, chronic total occlusion, thrombus, calcification, and LVEF were restricted to admissions classified as having obstructive coronary artery disease. The STEMI analysis was exploratory, and its detailed angiographic comparisons were similarly restricted to admissions with obstructive STEMI.

Admissions without a valid calendar year contributed to the overall analysis but not to calendar‐year summaries. No mortality, readmission, or other longitudinal outcome was available; the study therefore did not assess the prognostic “SMuRF‐less paradox.”

### 2.6 Missing Data and Data Quality

No missing values were imputed. Analyses used available observations for each variable, with the relevant analysis‐set denominator reported wherever it could be established. For some binary registry fields, the coding structure did not consistently distinguish a confirmed negative finding from an unrecorded entry; these variables are therefore described as “recorded” and interpreted cautiously.

### 2.7 Statistical Analysis

Continuous variables were summarized as mean ± standard deviation and compared between SMuRF‐less and SMuRF‐positive admissions using Welch's two‐sample *t* test. Standardized mean differences were expressed as Cohen's *d*, calculated with the SMuRF‐positive group as the reference.

Categorical variables were summarized as counts and percentages and compared using the *χ* ^2^ test, with Yates continuity correction for 2 × 2 comparisons. Effect estimates for binary outcomes were expressed as prevalence ratios comparing SMuRF‐less with SMuRF‐positive admissions, together with 95% confidence intervals calculated on the logarithmic scale. No comparative test or effect estimate was applied to the four variables defining SMuRF status because their absence in the SMuRF‐less group was imposed by definition.

All tests were two‐sided, and *p* < 0.05 was considered statistically significant. Analyses were unadjusted; no multivariable modelling, correction for possible clustering of repeat admissions, or adjustment for multiple comparisons was performed. Given the large sample and the exploratory nature of several comparisons, interpretation emphasized the magnitude and precision of effect estimates rather than statistical significance alone.

Analyses were conducted using Python version 3.12.13 (Python Software Foundation), pandas version 2.2.3, and SciPy version 1.17.0.

### 2.8 Ethical Considerations

The study was approved by the Ethical Review Committee of Ibrahim Cardiac Hospital & Research Institute, Dhaka, Bangladesh (approval no. ICHRI/Research/ERC/2026/02). The requirement for individual informed consent was waived for this retrospective use of routinely collected clinical data. Data were de‐identified before analysis, and the study was conducted in accordance with the Declaration of Helsinki.

## 3 Results

### 3.1 Cohort Characteristics and Frequency of SMuRF‐Less Admissions

The analytic cohort comprised 51,864 admissions with a matched cardiac catheterization record between January 2005 and December 2024. Under the primary registry‐based definition, 3172 admissions (6.1%) were classified as SMuRF‐less and 48,692 (93.9%) as SMuRF‐positive. The corresponding prevalence was 6.5% (3348/51,864) when dyslipidaemia was based only on its documented diagnostic flag and 1.6% (805/51,864) under the statin‐inclusive sensitivity definition (Table 1).

**Table 1:** Frequency of admissions classified as SMuRF‐less under alternative registry‐based definitions, overall and by procedural category.

Cohort or procedural category	Admissions, *N*	Documented‐only definition, *n* (%)	Primary definition, *n* (%)	Statin‐inclusive sensitivity definition, *n* (%)
Entire analytic cohort	51,864	3348 (6.5)	3172 (6.1)	805 (1.6)
CAG only	30,932	2193 (7.1)	2101 (6.8)	709 (2.3)
CAG and PCI during the same admission	14,220	761 (5.4)	686 (4.8)	41 (0.3)
Staged CAG and PCI	1377	80 (5.8)	77 (5.6)	4 (0.3)
PCI only	4551	236 (5.2)	234 (5.1)	40 (0.9)
Primary PCI	756	72 (9.5)	68 (9.0)	6 (0.8)
Procedural category not otherwise classified^a^	28	6 (21.4)	6 (21.4)	5 (17.9)
Diagnosis‐based subgroup^b^				
STEMI	6635	433 (6.5)	355 (5.4)	20 (0.3)

Abbreviations: CAG, coronary angiography; PCI, percutaneous coronary intervention; SMuRF, standard modifiable cardiovascular risk factor; STEMI, ST‐elevation myocardial infarction.

^a^This residual category comprises admissions not assigned to one of the five specified procedural categories.

^b^STEMI is a diagnosis‐based subgroup that overlaps the procedural categories.

The five reported procedural categories accounted for 51,836 admissions; 28 admissions, including six classified as SMuRF‐less, had no reported procedural category. Among the five categories, the proportion classified as SMuRF‐less under the primary definition was 6.8% for CAG‐only admissions, 4.8% for CAG and PCI during the same admission, 5.6% for staged CAG and PCI, 5.1% for PCI‐only admissions, and 9.0% for primary PCI admissions. Among the 6635 admissions presenting with STEMI, 355 (5.4%) were classified as SMuRF‐less.

Calendar year was available for 51,860 admissions; four SMuRF‐positive admissions had no valid calendar‐year entry. The annual distribution is presented descriptively in Supplementary Table S1. No formal temporal‐trend analysis was undertaken. All comparisons reported below are unadjusted and use admissions as the unit of analysis.

### 3.2 Recorded Baseline Characteristics

Admissions classified as SMuRF‐less had a lower reported mean age than SMuRF‐positive admissions (52.0 ± 12.7 vs. 56.4 ± 10.5 years; Cohen's *d* = −0.41) and a lower reported mean body mass index (25.0 ± 3.8 vs. 25.9 ± 3.8 kg/m^2^; Cohen's *d* = −0.25) (Table 2). Male sex was recorded in 63.9% and 73.0%, respectively (prevalence ratio [PR], 0.87; 95% confidence interval [CI], 0.85–0.90). Chronic kidney disease was less frequently recorded among SMuRF‐less admissions (4.7% vs. 16.0%; PR, 0.29; 95% CI, 0.25–0.34).

**Table 2:** Recorded baseline characteristics according to the primary registry‐based SMuRF classification.

Characteristic	SMuRF‐less admissions, *n* = 3172	SMuRF‐positive admissions, *n* = 48,692	Unadjusted effect estimate
Non‐defining characteristics			
Age, years	52.0 ± 12.7	56.4 ± 10.5	Cohen's d = −0.41
Male sex	2027 (63.9)	35,563 (73.0)	PR 0.87 (95% CI 0.85–0.90)
BMI, kg/m^2^	25.0 ± 3.8	25.9 ± 3.8	Cohen's d = −0.25
Chronic kidney disease	148 (4.7)	7795 (16.0)	PR 0.29 (95% CI 0.25–0.34)
Components defining SMuRF status			
Documented hypertension	0 by definition	38,710 (79.5)	Not applicable
Documented diabetes	0 by definition	32,728 (67.2)	Not applicable
Dyslipidaemia under the primary definition	0 by definition	27,909 (57.3)	Not applicable
Documented smoking	0 by definition	10,601 (21.8)	Not applicable

*Note:* Prevalence‐ratio entries are presented with 95% confidence intervals.

Abbreviations: CAD, coronary artery disease; CI, confidence interval; LVEF, left ventricular ejection fraction; PR, prevalence ratio; SMuRF, standard modifiable cardiovascular risk factor; STEMI, ST‐elevation myocardial infarction.

By construction, none of the admissions classified as SMuRF‐less had a recorded qualifying SMuRF under the primary definition. Among SMuRF‐positive admissions, hypertension was recorded in 79.5%, diabetes in 67.2%, dyslipidaemia under the primary definition in 57.3%, and smoking in 21.8%. These characteristics were not mutually exclusive.

### 3.3 Coronary Angiographic Phenotype

A classifiable coronary angiographic phenotype was available for 2927 of 3172 SMuRF‐less admissions (92.3%) and 44,272 of 48,692 SMuRF‐positive admissions (90.9%). The remaining 4665 admissions did not have a classifiable phenotype.

Among admissions with a classifiable phenotype, normal coronary arteries were more frequently observed in the SMuRF‐less group than in the SMuRF‐positive group (24.9% vs. 10.7%; PR, 2.34; 95% CI, 2.18–2.50; *p *< 0.001) (Table 3). The prevalence of non‐obstructive disease was similar between the groups (18.5% vs. 18.1%; PR, 1.02; 95% CI, 0.94–1.10; *p* = 0.650). Obstructive CAD was less frequently observed among SMuRF‐less admissions (56.6% vs. 71.2%; PR, 0.79; 95% CI, 0.77–0.82; *p* < 0.001).

**Table 3:** Coronary angiographic phenotype according to the primary registry‐based SMuRF classification.

Variable	SMuRF‐less	SMuRF‐positive	Unadjusted PR (95% CI)	*p* value
Classifiable angiographic phenotype, *n*/*N* (%)	2927/3172 (92.3)	44,272/48,692 (90.9)	—	—
Among admissions with a classifiable phenotype				
Normal coronary arteries	730 (24.9)	4723 (10.7)	2.34 (2.18–2.50)	< 0.001
Non‐obstructive disease	541 (18.5)	8027 (18.1)	1.02 (0.94–1.10)	0.650
Obstructive CAD	1,656 (56.6)	31,522 (71.2)	0.79 (0.77–0.82)	< 0.001
Among admissions with obstructive CAD				
Single‐vessel disease	826 (49.9)	13,184 (41.8)	1.19 (1.13–1.25)	< 0.001
Double‐vessel disease	514 (31.0)	10,901 (34.6)	0.90 (0.83–0.97)	0.003
Triple‐vessel disease	308 (18.6)	7312 (23.2)	0.80 (0.72–0.89)	< 0.001
Not assigned to an SVD/DVD/TVD category^a^	8 (0.5)	125 (0.4)	—	—
Multivessel disease^b^	822 (49.6)	18,213 (57.8)	0.86 (0.82–0.90)	< 0.001
Other angiographic and functional features among admissions with obstructive CAD				
Left‐main disease	82 (5.0)	1753 (5.6)	0.89 (0.72–1.10)	0.316
Severe LAD disease	1209 (73.0)	23,376 (74.2)	0.98 (0.95–1.01)	0.311
Severe LCx disease	647 (39.1)	14,579 (46.3)	0.84 (0.79–0.90)	< 0.001
Severe RCA disease	922 (55.7)	18,967 (60.2)	0.93 (0.89–0.97)	< 0.001
Chronic total occlusion	253 (15.3)	4,912 (15.6)	0.98 (0.87–1.10)	0.765
Recorded intracoronary thrombus	88 (5.3)	1,016 (3.2)	1.65 (1.33–2.04)	< 0.001
Recorded coronary calcification	132 (8.0)	3080 (9.8)	0.82 (0.69–0.96)	0.018
Reduced LVEF, ≤ 40%	291 (17.6)	5460 (17.3)	1.01 (0.91–1.13)	0.818

Abbreviations: CAD, coronary artery disease; CI, confidence interval; DVD, double‐vessel disease; LAD, left anterior descending artery; LCx, left circumflex artery; LVEF, left ventricular ejection fraction; PR, prevalence ratio; RCA, right coronary artery; SMuRF, standard modifiable cardiovascular risk factor; SVD, single‐vessel disease; TVD, triple‐vessel disease.

^a^These admissions were retained in the obstructive‐CAD denominator but were not assigned to a single‐, double‐, or triple‐vessel disease category.

^b^Multivessel disease comprises double‐ and triple‐vessel disease and is therefore not an additional mutually exclusive category.

### 3.4 Angiographic Features among Admissions With Obstructive CAD

The obstructive‐CAD analysis included 1,656 SMuRF‐less and 31,522 SMuRF‐positive admissions. Single‐vessel disease was more frequent among SMuRF‐less admissions (49.9% vs. 41.8%; PR, 1.19; 95% CI, 1.13–1.25; *p* < 0.001). Conversely, multivessel disease was less frequent (49.6% vs. 57.8%; PR, 0.86; 95% CI, 0.82–0.90; *p* < 0.001), including both double‐vessel disease (31.0% vs. 34.6%; PR, 0.90; 95% CI, 0.83–0.97; *p* = 0.003) and triple‐vessel disease (18.6% vs. 23.2%; PR, 0.80; 95% CI, 0.72–0.89; *p* < 0.001). Eight SMuRF‐less and 125 SMuRF‐positive admissions with obstructive CAD were not assigned to a single‐, double‐, or triple‐vessel category.

Severe left circumflex disease (39.1% vs. 46.3%; PR, 0.84; 95% CI, 0.79–0.90; *p* < 0.001) and severe right coronary artery disease (55.7% vs. 60.2%; PR, 0.93; 95% CI, 0.89–0.97; *p* < 0.001) were less frequently recorded among SMuRF‐less admissions. There was no clear difference in left‐main disease (5.0% vs. 5.6%; PR, 0.89; 95% CI, 0.72–1.10; *p* = 0.316), severe left anterior descending artery disease (73.0% vs. 74.2%; PR, 0.98; 95% CI, 0.95–1.01; *p* = 0.311), or chronic total occlusion (15.3% vs. 15.6%; PR, 0.98; 95% CI, 0.87–1.10; *p* = 0.765).

Recorded intracoronary thrombus was more frequent among SMuRF‐less admissions (5.3% vs. 3.2%; PR, 1.65; 95% CI, 1.33–2.04; *p* < 0.001), whereas recorded coronary calcification was less frequent (8.0% vs. 9.8%; PR, 0.82; 95% CI, 0.69–0.96; *p* = 0.018). The proportion with a recorded left ventricular ejection fraction of 40% or lower was similar between the groups (17.6% vs. 17.3%; PR, 1.01; 95% CI, 0.91–1.13; *p* = 0.818).

### 3.5 Exploratory STEMI Subgroup Analysis

The exploratory STEMI subgroup comprised 355 SMuRF‐less and 6,280 SMuRF‐positive admissions (Table 4). Reported mean age was similar between the groups (53.9 ± 11.5 vs 54.9 ± 10.7 years; Cohen's *d* = −0.09; *p* = 0.105), as was the proportion with obstructive CAD (89.6% vs 90.6%; PR, 0.99; 95% CI, 0.95–1.03; *p* = 0.582). Male sex was recorded in 87.9% and 84.4%, respectively (PR, 1.04; 95% CI, 1.00–1.08; *p* = 0.094). A left ventricular ejection fraction of 40% or lower was recorded in 29.6% of SMuRF‐less and 29.7% of SMuRF‐positive STEMI admissions (PR, 0.99; 95% CI, 0.84–1.17; *p* = 0.999).

**Table 4:** Exploratory analysis of STEMI admissions according to the primary registry‐based SMuRF classification.

Variable	SMuRF‐less, *n* = 355	SMuRF‐positive, *n* = 6280	Unadjusted effect estimate	*p* value
Age, years	53.9 ± 11.5	54.9 ± 10.7	Cohen's d = −0.09	0.105
Male sex	312 (87.9)	5,303 (84.4)	PR 1.04 (1.00–1.08)	0.094
Obstructive CAD	318 (89.6)	5690 (90.6)	PR 0.99 (0.95–1.03)	0.582
Reduced LVEF, ≤ 40%	105 (29.6)	1867 (29.7)	PR 0.99 (0.84–1.17)	0.999
Among admissions with obstructive STEMI				
Multivessel disease	135 (42.5)	2961 (52.0)	PR 0.82 (0.72–0.93)	0.001
Left‐main disease	5 (1.6)	152 (2.7)	PR 0.59 (0.24–1.42)	0.310
Chronic total occlusion	33 (10.4)	599 (10.5)	PR 0.99 (0.71–1.37)	> 0.999
Recorded intracoronary thrombus	36 (11.3)	506 (8.9)	PR 1.27 (0.93–1.75)	0.171

*Note:* Prevalence‐ratio entries are presented with 95% confidence intervals.

Abbreviations: CAD, coronary artery disease; CI, confidence interval; LVEF, left ventricular ejection fraction; PR, prevalence ratio; SMuRF, standard modifiable cardiovascular risk factor; STEMI, ST‐elevation myocardial infarction.

Among admissions with obstructive STEMI, multivessel disease was less frequent in the SMuRF‐less group (42.5% vs. 52.0%; PR, 0.82; 95% CI, 0.72–0.93; *p* = 0.001). Recorded intracoronary thrombus was numerically more frequent (11.3% vs. 8.9%), although the estimate was imprecise and compatible with no difference (PR, 1.27; 95% CI, 0.93–1.75; *p* = 0.171). No clear between‐group differences were observed in left‐main disease or chronic total occlusion.

## 4 Discussion

### 4.1 Principal Findings

In this 20‐year registry‐based analysis of 51,864 catheterized admissions, 6.1% were classified as SMuRF‐less under the primary definition. The estimated frequency changed only modestly when dyslipidaemia was based exclusively on its documented diagnostic flag but fell substantially when lipid‐lowering treatment was also regarded as evidence of dyslipidaemia. Admissions classified as SMuRF‐less were younger and had lower recorded body mass index and chronic kidney disease prevalence than SMuRF‐positive admissions.

The angiographic findings suggest a lower overall burden of obstructive coronary disease among SMuRF‐less admissions. Among admissions with a classifiable phenotype, normal coronary arteries were more than twice as frequent, while obstructive CAD was less frequent; the prevalence of non‐obstructive disease was similar between the groups. Among admissions with obstructive CAD, SMuRF‐less status was associated with more single‐vessel and less multivessel disease, together with less frequent severe left circumflex and right coronary artery involvement. Left‐main disease, severe left anterior descending artery involvement, chronic total occlusion and reduced LVEF were similar between the groups. Recorded intracoronary thrombus was more frequent and coronary calcification less frequent among SMuRF‐less admissions, although the thrombus difference was smaller and statistically inconclusive in the exploratory STEMI subgroup. Collectively, these findings describe an unadjusted admission‐level pattern of less extensive angiographic disease, not an independently established or mechanistically distinct coronary phenotype; the reported prevalence ratios should therefore not be interpreted as causal effects of SMuRF status.

### 4.2 Frequency and Definition of SMuRF‐Less Status

The 6.1% overall frequency observed in this study should not be compared directly with estimates derived exclusively from ACS or first‐presentation MI cohorts because the present population encompassed the broader spectrum of admissions undergoing cardiac catheterization. Within the STEMI subgroup, 5.4% of admissions were classified as SMuRF‐less. This is lower than the 12.9% pooled estimate reported for STEMI in a global meta‐analysis and the 8.6% reported in a multiethnic Asian ACS cohort [6, 13]. It is also lower than the 15% reported among first‐episode ACS presentations at a tertiary cardiac centre in Pakistan [14]. Such variation is unsurprising because published estimates differ according to population selection, prior cardiovascular disease, referral pathways, risk‐factor definitions, availability of laboratory measurements and whether undocumented factors are assumed to be absent.

These findings should also be interpreted alongside the broader international literature: SWEDEHEART evaluated SMuRF‐less status principally in first‐presentation acute myocardial infarction and identified adverse early outcomes, whereas INTERHEART demonstrated the younger age of first myocardial infarction among South Asian populations [7, 10]. Together, these observations reinforce the importance of considering clinical setting, population characteristics and risk‐factor ascertainment when comparing SMuRF‐less cohorts.

The sensitivity analyses within the present study illustrate this definitional dependence directly. Incorporating available lipid measurements into the documented dyslipidaemia flag reduced the SMuRF‐less estimate only from 6.5% to 6.1%, whereas additionally treating lipid‐lowering therapy as evidence of dyslipidaemia reduced it to 1.6%. The latter estimate should not be interpreted as a more accurate prevalence because treatment may have been initiated for established or newly identified coronary disease rather than for known pre‐admission dyslipidaemia. Instead, the large change demonstrates why medication use cannot be substituted uncritically for antecedent risk‐factor status. More generally, “SMuRF‐less” in this study denotes an absence of documented qualifying factors within routinely collected records, not a prospectively verified absence of cardiovascular susceptibility.

The alternative dyslipidaemia definitions provide a sensitivity analysis for one component of this ascertainment problem, but they cannot eliminate misclassification arising from incomplete documentation of hypertension, diabetes or smoking.

### 4.3 Interpretation of the Angiographic Phenotype

The greater frequency of normal coronary arteries and lower frequency of obstructive disease among SMuRF‐less admissions are broadly consistent with previous evidence that this population is heterogeneous. In a propensity‐score‐matched ACS study, Papazoglou et al. [15] found that SMuRF‐less status was associated with a greater probability of a zero SYNTAX score, although the severity distribution among those with a non‐zero score was similar between groups. The present findings extend that observation across a much broader catheterization population: SMuRF‐less admissions were more likely to have normal arteries, but more than half of those with a classifiable phenotype nevertheless had obstructive CAD.

Among admissions with obstruction, the higher frequency of single‐vessel disease and lower frequency of multivessel disease, together with less frequent recorded calcification, may indicate a lower accumulated atherosclerotic burden among SMuRF‐less admissions. This interpretation is compatible with serial intravascular‐ultrasound data showing lower baseline plaque burden among individuals without SMuRFs, although subsequent plaque progression was comparable with that of individuals who had conventional risk factors [16]. Studies of young patients with STEMI have characterized the clinical and angiographic profiles of those without SMuRFs; one angiographic study reported shorter and less complex lesions in this group [17, 18]. These comparisons nevertheless require caution. In the present study, SMuRF‐less admissions were younger by more than 4 years, and age, sex, clinical presentation and registry era were not adjusted for. The observed anatomical differences therefore cannot be attributed independently to SMuRF status.

One possible explanation for the higher recorded frequency of intracoronary thrombus, particularly when considered alongside less frequent calcification, is a greater relative contribution from acute thrombotic presentation than from longstanding calcific atherosclerotic burden. Reports of poorer preprocedural coronary flow among SMuRF‐less patients with STEMI provide limited external support for this possibility [19]. However, the present data do not establish such a mechanism. The thrombus association was attenuated and statistically inconclusive within the STEMI subgroup, the timing and indication for angiography varied across the cohort, and the comparisons were unadjusted. Moreover, conventional angiography cannot determine plaque composition or reliably distinguish plaque rupture, plaque erosion, embolism, spontaneous coronary dissection or other mechanisms of coronary occlusion. Without optical coherence tomography, intravascular ultrasonography or independent image adjudication, the thrombus finding should remain hypothesis‐generating and must not be interpreted as evidence of plaque erosion or a distinct acute pathological process.

The exploratory STEMI analysis further tempers any claim of a uniformly distinct SMuRF‐less phenotype. Obstructive disease and reduced LVEF occurred at similar frequencies in the two groups, and clear differences were not observed in left‐main disease, chronic total occlusion or intracoronary thrombus. Multivessel disease remained less frequent among SMuRF‐less admissions, but less extensive coronary anatomy should not be equated with lower clinical risk. Associations between SMuRF‐less status and outcomes have varied across STEMI, NSTEMI, broader ACS, and stable or revascularized CAD cohorts [6, 7, 8, 20, 21, 22, 23]. Some acute cohorts have reported adverse early outcomes despite fewer comorbidities, whereas stable‐CAD cohorts have shown more favourable longer‐term outcomes. Because the present study contained no mortality or follow‐up data, it cannot determine whether its anatomical findings translate into either favourable or adverse prognosis.

### 4.4 Clinical and Research Implications

The findings reinforce two complementary clinical messages. First, conventional modifiable risk factors remain strongly associated with the burden and extent of coronary atherosclerosis at the population level. Second, their documented absence cannot safely be used to exclude clinically important coronary disease. Obstructive CAD was present in 56.6% of classifiable SMuRF‐less admissions and in 89.6% of SMuRF‐less STEMI admissions. Diagnostic evaluation and evidence‐based secondary prevention should therefore be guided by the clinical syndrome and established coronary disease rather than by the perceived reassurance of having no recorded SMuRF.

Prospective studies should establish SMuRF status through standardized blood‐pressure assessment, biochemical testing and detailed tobacco‐exposure history rather than relying primarily on diagnostic flags. Patient‐level linkage is needed to identify repeat admissions, while multivariable analyses should account for age, sex, presentation, treatment and calendar era. Evaluation of family history, lipoprotein(a), inflammatory and autoimmune disorders, genetic susceptibility and other potential pathways may help characterize residual risk, but these factors should be investigated systematically rather than presumed to explain disease in every SMuRF‐less individual. Longitudinal outcome assessment and intracoronary imaging would also be required to connect angiographic patterns with prognosis and underlying plaque biology.

### 4.5 Strengths and Limitations

The principal strengths of this study are its large sample, two decades of clinical practice and inclusion of a broad catheterization population rather than a narrowly selected ACS cohort. The Bangladeshi setting adds evidence from a region underrepresented in the SMuRF‐less literature. Linking discharge and catheterization records allowed clinical classification to be examined alongside detailed coronary anatomy, while the alternative dyslipidaemia definitions demonstrated the sensitivity of estimated frequency to risk‐factor ascertainment.

Several limitations require equal emphasis. First, this was a retrospective study from a single tertiary cardiac centre and included only admissions selected for catheterization. Its findings therefore cannot estimate SMuRF‐less prevalence in the Bangladeshi population or be generalized to people who were not referred for invasive assessment. Second, the unit of analysis was the admission; repeat admissions belonging to the same individual could not be reliably excluded and may therefore have been retained. The resulting lack of statistical independence may have narrowed confidence intervals and given disproportionate weight to individuals with multiple procedures.

Third, SMuRF classification depended substantially on routine documentation. Hypertension, diabetes and smoking were not prospectively excluded, missing lipid values were not imputed, and an unrecorded binary field could not always be distinguished from a true negative finding. Misclassification is therefore likely, particularly if access to prior diagnoses or laboratory testing differed between admissions. In addition, the four‐factor SMuRF construct does not encompass all potentially modifiable cardiovascular exposures. Factors such as adiposity, physical inactivity, dietary exposures and forms of tobacco exposure not represented by the recorded smoking field may therefore contribute to residual cardiovascular risk and to differences between the groups.

Fourth, the 20‐year period spanned two registry systems and major changes in diagnostic practice, referral patterns, preventive treatment and data completeness. Calendar‐year patterns cannot consequently be interpreted as epidemiological trends.

Fifth, variable‐specific completeness could not be fully established from the supplied data. Twenty‐eight admissions were not assigned to one of the five reported procedural categories, 4665 lacked a classifiable angiographic phenotype, and 133 admissions with obstructive CAD were not assigned to a single‐, double‐ or triple‐vessel category. Recorded thrombus, calcification and other binary angiographic findings may also have included unrecorded observations coded as absent. Sixth, all comparisons were unadjusted, no correction was made for possible clustering or multiple testing, and several analyses, particularly the STEMI subgroup, were exploratory. Differences in age, sex, clinical presentation and registry era may account for part of the observed phenotype. Finally, angiographic images were not independently re‐adjudicated, intracoronary imaging was unavailable, and no longitudinal clinical outcomes were analysed. The study can therefore characterize recorded coronary anatomy but cannot establish biological mechanisms, causal effects or the prognostic SMuRF‐less paradox.

## 5 Conclusion

In this registry‐based analysis of 51,864 catheterized admissions in Bangladesh, 6.1% were classified as SMuRF‐less under the primary definition, although the estimated frequency was sensitive to how dyslipidaemia was ascertained. SMuRF‐less admissions generally had a lower recorded angiographic burden, with more normal coronary arteries, less obstructive CAD and less multivessel disease than SMuRF‐positive admissions. Nevertheless, obstructive CAD remained present in more than half of classifiable SMuRF‐less admissions and in nearly nine in ten SMuRF‐less STEMI admissions. Within this catheterized cohort, the absence of documented conventional risk factors did not exclude substantial obstructive disease and should not be considered reassuring when clinical evaluation indicates possible coronary disease. Because these findings were derived from routinely recorded, admission‐level and unadjusted data, prospective patient‐level studies with standardized risk‐factor ascertainment and longitudinal follow‐up are needed to determine their independent and prognostic significance.

## Author Contributions

**Md Mahbub Hasan:** project administration, supervision, writing – review and editing, writing – original draft, investigation, visualization, formal analysis. **M. Maksumul Haq:** project administration, supervision, investigation, conceptualization, formal analysis, data curation, writing – review and editing. **CM Shaheen Kabir:** project administration, investigation. **Mashhud Zia Chowdhury:** investigation, project administration. **Saidur Rahman Khan:** investigation, project administration. **Md Hasanur Rahman:** investigation, project administration. **Sahela Nasrin:** investigation, project administration. **Isha Abdullah Ali:** investigation. **Khondoker Md Ferdoush Siraj:** investigation. **Arifur Rahman:** investigation. **Sayem Bin Latif:** investigation.

## Funding

The authors have nothing to report.

## Conflicts of Interest

The authors declare no conflicts of interest.

## Declaration of Generative AI and AI‐Assisted Technologies in the Writing Process

During the preparation of this work, the authors used ChatGPT (OpenAI) for language editing and manuscript formatting assistance. After using this tool, the authors reviewed and edited the content as needed and take full responsibility for the content of the published article.

## Transparency Statement

The lead and corresponding author, Md Mahbub Hasan, affirms that this manuscript is an honest, accurate, and transparent account of the study being reported; that no important aspects of the study have been omitted; and that any discrepancies from the study as planned (and, if relevant, registered) have been explained.

## Supporting information


Supporting File

## Data Availability

The data that support the findings of this study are not publicly available because access is restricted by institutional policy.
