# Supplementary material for: Absence of Documented Standard Modifiable Cardiovascular Risk Factors Among Catheterized Admissions in Bangladesh: Frequency and Coronary Angiographic Phenotype in a 20‐Year Registry Study
Source: Health Sci Rep. 2026 Nov 4;9(11):e73466. doi: 10.1002/hsr2.73466 (PMC13732564; doi:10.1002/hsr2.73466)
Supplement: Supplementary file 1 [file HSR2-9-e73466-s001.docx]

**Supplementary Table S1. Calendar-year distribution of admissions classified as SMuRF-less under the primary registry-based definition**

| **Calendar year** | **Admissions, N** | **SMuRF-less, n (%)** |
| --- | --- | --- |
| **2005** | 246 | 13 (5.3) |
| **2006** | 477 | 24 (5.0) |
| **2007** | 774 | 33 (4.3) |
| **2008** | 1,333 | 68 (5.1) |
| **2009** | 1,554 | 63 (4.1) |
| **2010** | 1,736 | 68 (3.9) |
| **2011** | 2,044 | 117 (5.7) |
| **2012** | 2,115 | 131 (6.2) |
| **2013** | 1,868 | 87 (4.7) |
| **2014** | 2,158 | 151 (7.0) |
| **2015** | 2,343 | 225 (9.6) |
| **2016** | 2,856 | 241 (8.4) |
| **2017** | 3,119 | 223 (7.1) |
| **2018** | 2,881 | 200 (6.9) |
| **2019** | 2,986 | 200 (6.7) |
| **2020** | 3,095 | 167 (5.4) |
| **2021** | 4,283 | 242 (5.7) |
| **2022** | 5,756 | 368 (6.4) |
| **2023** | 6,315 | 349 (5.5) |
| **2024** | 3,921 | 202 (5.2) |
| **Calendar year unavailable¹** | 4 | 0 |
| **Overall** | 51,864 | 3,172 (6.1) |

¹Calendar year was unavailable for four SMuRF-positive admissions. Annual percentages are descriptive; no formal trend test was performed because documentation completeness differed between the two registry systems.

Abbreviation: SMuRF, standard modifiable cardiovascular risk factor.
